# Frailty mediates the relationship between kidney function measures and all-cause mortality among middle-aged and older adults: Findings from stratified analysis

**DOI:** 10.1097/MD.0000000000049214

**Published:** 2026-06-19

**Authors:** Bin Li, Yalin Niu, Baosai Lu, Yuewei Yin, Chenming Zhao, Wei Li

**Affiliations:** a Department of Urology, The Second Hospital of Hebei Medical University, Shijiazhuang, Hebei Province, China.

**Keywords:** adults, frailty, kidney function, mortality

## Abstract

Chronic kidney disease (CKD) and frailty are prevalent conditions in middle-aged and older adults; both are independently associated with an increased mortality risk. However, the interrelationship between kidney function, frailty, and mortality in individuals aged 45 years and older remains incompletely understood. We conducted a cross-sectional analysis of frailty prevalence and a longitudinal analysis of mortality outcomes using data from 9079 participants aged ≥45 years from the National Health and Nutrition Examination Survey (2007–2018). Kidney function was assessed by estimated glomerular filtration rate (eGFR), albumin-to-creatinine ratio (ACR), and CKD status. Frailty was defined using a modified Fried phenotype. All-cause mortality was determined through National Death Index linkage. Frailty prevalence was 15.7%. CKD prevalence, lower eGFR, and higher ACR were associated with higher odds of frailty. During a median follow-up of 77 months (interquartile range: 35–117 months), 18.8% of participants died. Impaired kidney function, as defined by reduced eGFR, elevated ACR, or the presence of CKD, was associated with a higher mortality risk, particularly among frail participants. Frailty mediated approximately 4% of the association between ACR/CKD and mortality. Impaired kidney function is significantly associated with frailty. The association between impaired kidney function and mortality differed descriptively across frailty strata. Frailty mediated a portion of this association, highlighting its importance in the clinical management of individuals with impaired kidney function.

**Strengths and limitations of the study**

1. The cross-sectional design prevents causal inference.
2. Some analyses rely on self-reported data, introducing potential bias.
3. Prospective studies are needed to confirm these findings.

## 1. Introduction

Chronic kidney disease (CKD) is a significant public health concern, particularly among middle-aged and older adults. The prevalence of CKD increases with age, contributing to a spectrum of adverse health outcomes including anemia, cardiovascular events, diabetes, increased hospitalization rates, and elevated mortality risk in this population.^[1–4]^ CKD, characterized by progressive loss of renal function, can advance to end-stage renal disease, necessitating renal replacement therapy.^[5]^ However, even in its earlier stages, CKD is associated with a range of comorbidities including cardiovascular disease, cognitive impairment, and frailty.^[6]^

Frailty, a geriatric syndrome defined by diminished physiological reserves and heightened vulnerability to stressors, has emerged as a crucial predictor of adverse outcomes in older adults, including those with CKD.^[7]^ This syndrome, encompassing physical weakness, slowed motor performance, fatigue, and unintentional weight loss, significantly increases the risk of falls, hospitalization, and mortality.^[8]^ Recent evidence highlights that the prevalence of frailty in CKD patients ranges from 7% in community-dwelling individuals to over 70% in dialysis populations, far exceeding rates in the general elderly population.^[9]^ Impaired kidney function may accelerate the development of frailty through various mechanisms, including chronic inflammation, malnutrition, and reduced physical activity.^[8,10,11]^ The interplay between kidney dysfunction and frailty is particularly concerning, as these conditions both independently contribute to poor health outcomes, including increased mortality risk.^[12]^

Most existing studies, including the National Health and Nutrition Examination Survey (NHANES) analysis by Zhang et al. (2020), have focused primarily on the direct association between frailty and mortality in CKD patients.^[13,14]^ However, our study examines frailty as a mediator of the kidney function-mortality relationship and descriptively explores whether this association differs across frailty strata. Furthermore, we utilize more recent NHANES cycles (2007–2018) and employ sophisticated statistical approaches to quantify mediation effects. The NHANES offers an ideal platform for investigating these complex relationships. Leveraging this robust dataset, our study aims to elucidate the intricate relationships among kidney function, frailty, and mortality in middle-aged and older adults. Specifically, this investigation seeks to: examine the associations between various measures of kidney function and frailty; evaluate the impact of kidney function on mortality risk, stratified by frailty status; and explore the potential mediating effect of frailty on the relationship between kidney function and mortality.

## 2. Materials and methods

### 2.1. Study population and design

This study utilized data from the NHANES, a cross-sectional, nationally representative survey conducted by the National Center for Health Statistics of the Centers for Disease Control and Prevention. NHANES employs a complex, multistage probability sampling design to assess the health and nutritional status of the noninstitutionalized US civilian population. The survey protocol, which received approval from the NCHS Research Ethics Review Board, comprises in-home interviews followed by physical examinations and laboratory tests at Mobile Examination Centers. All participants provided written informed consent. Detailed methodological information is publicly available (https://www.cdc.gov/nchs/nhanes.htm). The whole study was performed according to the Declaration of Helsinki.

**Figure 1.**
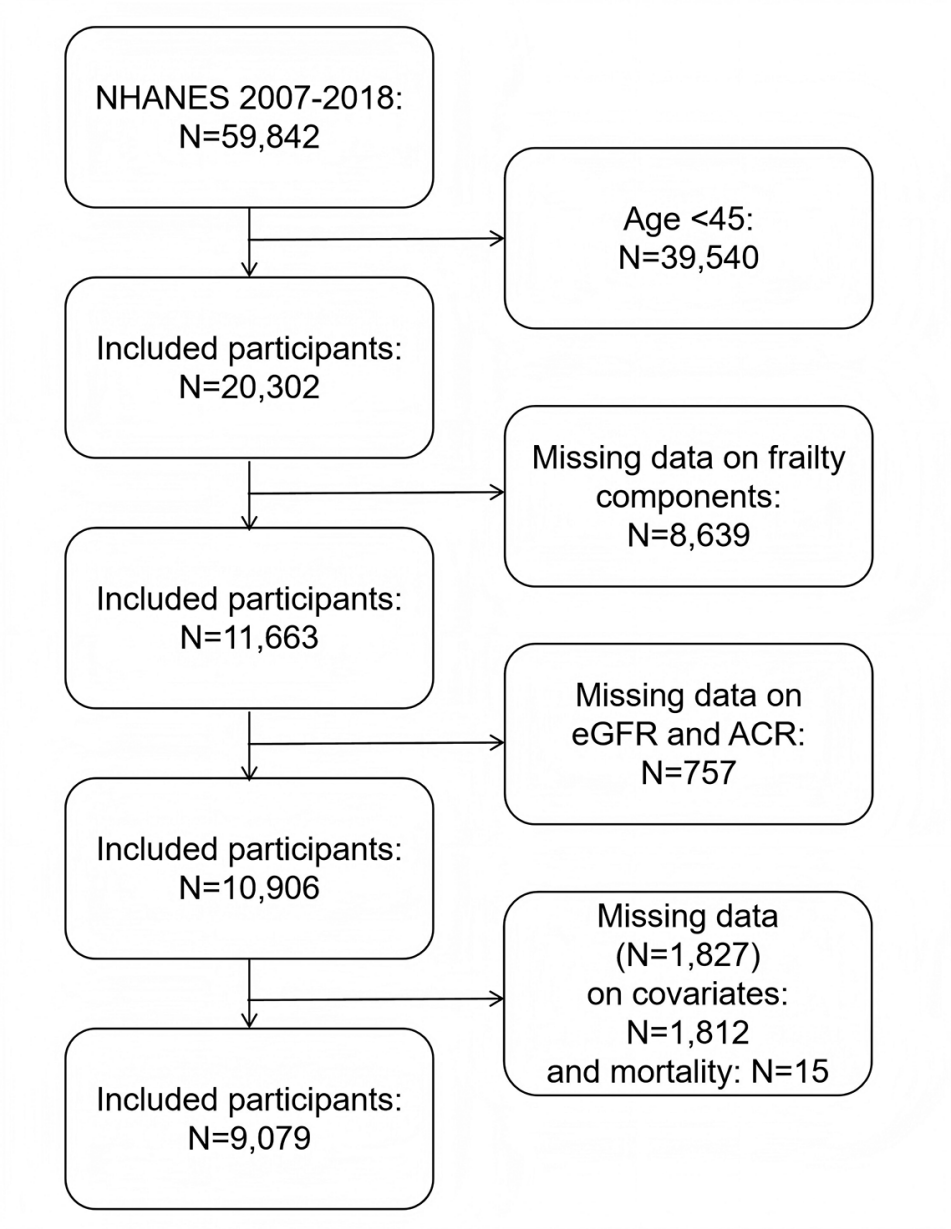
Flow chart of participant selection (NHANES 2007–2018). NHANES = National Health and Nutrition Examination Survey.

We aggregated data from 6 consecutive NHANES cycles (2007–2018), initially encompassing 59,842 individuals. While baseline measurements of kidney function and frailty were cross-sectional, mortality outcomes were assessed longitudinally through December 31, 2019, providing a median follow-up time of 77 months (interquartile range: 35–117 months). Our analysis focused on 20,302 adults aged 45 years or older. We sequentially excluded participants with incomplete data on frailty components (n = 8639) to ensure consistent measurement of our key exposure variable, missing estimated glomerular filtration rate (eGFR) or albumin-to-creatinine ratio (ACR) measurements (n = 757), incomplete covariate information (n = 1812), and missing mortality data (n = 15). Covariates included age, sex, race/ethnicity, education, marital status, diabetes, hypertension, high-density lipoprotein, total cholesterol, serum potassium, and serum sodium. The final analytical sample comprised 9079 participants. Figure 1 presents a detailed flowchart of the participant selection process.

### 2.2. Kidney function

This study employed 3 indicators to comprehensively evaluate kidney function: eGFR, ACR, and the presence of CKD. The eGFR was calculated using the 2021 Chronic Kidney Disease Epidemiology Collaboration (CKD-EPI) equation, which incorporates serum creatinine levels measured via the enzymatic method.^[15,16]^ Urine albumin concentrations were determined through solid-phase fluorescence immunoassay, while urine creatinine was quantified using the modified Jaffé kinetic method. The ACR was computed from these urine measurements. CKD was defined based on established criteria: a urinary ACR exceeding 30 mg/g or an eGFR below 60 mL/min/1.73 m^2^.^[17]^ For analysis, we stratified eGFR into 3 categories: <60, 60 to 89, and 90 mL/min/1.73 m^2^ or greater (reference category).^[18]^ Similarly, ACR was categorized into 3 groups: <30 (reference), 30 to 300, and >300 mg/g.^[19]^

### 2.3. Physical frailty

We assessed physical frailty using a modified version of the Fried frailty phenotype, adapted for use with NHANES data. This approach, validated in previous large-scale studies,^[20–22]^ utilizes self-reported data when direct physical measurements are unavailable.^[23]^ The 5 criteria for frailty were operationalized as follows: weakness: self-reported difficulty lifting or carrying 10 pounds; low physical activity: highest age-specific quintile of self-reported sedentary time; exhaustion: self-reported tiredness or lack of energy; slow walking speed: self-reported difficulty walking between rooms on the same floor; and unintentional weight loss: self-reported unintentional weight loss of 10 pounds or more in the past year, or low body mass index (<18.5 kg/m^2^). Participants meeting 3 or more criteria were classified as frail; those meeting <3 criteria as non-frail.

### 2.4. Mortality

All-cause mortality was determined using the 2019 public-use linked mortality files, which integrate data from the National Death Index (NDI) with NHANES participants’ records through December 31, 2019. The NDI, maintained by the National Center for Health Statistics, serves as a centralized repository of death record information from state vital statistics offices. Participant survival time was calculated from the date of the NHANES examination to either the date of death or the end of the follow-up period (December 31, 2019) for those who remained alive. The linkage between NHANES and NDI data was accomplished through a probabilistic matching process using unique study identifiers. This approach ensures comprehensive mortality ascertainment for the study cohort. Detailed information on the mortality linkage methodology is available at https://www.cdc.gov/nchs/ndi.

### 2.5. Covariates

We also incorporated a comprehensive set of covariates potentially associated with kidney function and frailty. These included sociodemographic characteristics, comorbidities, and biomedical parameters. Sociodemographic data were obtained from the NHANES questionnaire. Race/ethnicity was categorized as Mexican American, other Hispanic, non-Hispanic White, non-Hispanic Black, and other. Educational attainment was classified into 3 levels: less than high school, high school graduate (including general educational development equivalency), and more than high school education. Marital status was dichotomized as married/partnered or not married/partnered. Comorbidities included diabetes and hypertension, both assessed through self-reported physician diagnoses. Biomedical parameters comprised high-density lipoprotein cholesterol (HDL) and total cholesterol (TC), measured in mg/dL using an enzymatic method with the Roche Modular P chemistry analyzer at the University of Minnesota, Minneapolis. Detailed protocols for all laboratory measurements are available in the NHANES Laboratory Procedures Manual (https://wwwn.cdc.gov/nchs/nhanes/Default.aspx).

### 2.6. Statistical analysis

Descriptive statistics were calculated for all variables, with continuous measures reported as means (standard deviations) and categorical variables as counts (percentages). Baseline characteristics were compared using unweighted independent *t* tests for continuous variables and χ^2^ tests for categorical variables. Regression and mediation analyses were conducted without incorporating NHANES sampling weights, strata, or primary sampling units. This decision was based on the study’s primary objective: to examine internal associations and associational pathways between kidney function, frailty, and mortality within the analytic sample, rather than to generate nationally representative point estimates or prevalence figures. Accordingly, all findings are presented as associations observed in this cohort and should not be interpreted as nationally representative estimates for the U.S. population. Descriptive statistics (e.g., frailty prevalence) are presented as sample characteristics only.

The associations between kidney function measures and frailty were evaluated using multivariable logistic regression models. Separate models were constructed for eGFR, ACR, and CKD status as dependent variables. Variables such as ACR were already log-transformed in the NHANES dataset to account for skewness. All models were adjusted for demographic factors (age, sex, race/ethnicity, educational attainment, and marital status) and clinical variables (HDL, TC, diabetes, and hypertension).

To assess the relationship between kidney function measures and all-cause mortality, we employed multivariable Cox proportional hazards regression models, adjusting for the aforementioned covariates and frailty status. To ensure the validity of the Cox model, we tested the proportional hazards assumption using Schoenfeld residuals and graphical diagnostics. Time-dependent covariates were tested to confirm that their effects on mortality risk remained proportional over time. These diagnostic procedures confirmed that the model assumptions were satisfied. Hazard ratios (HRs) with 95% confidence intervals (CIs) were calculated. Subgroup analyses stratified by frailty status were also conducted to explore potential heterogeneity in the associations. These stratified analyses are descriptive only and are not intended to serve as formal tests of interaction, as no kidney function × frailty interaction term was included in the primary models. Accordingly, no claims of effect modification or statistical moderation are made. We conducted a mediation analysis using a counterfactual-based decomposition framework to examine whether baseline frailty accounted for part of the association between baseline kidney function measures (eGFR, ACR, and CKD status) and subsequent mortality. Because both the exposure and mediator were measured at baseline, this analysis was intended to describe associational decomposition rather than causal mediation. Frailty was modeled using logistic regression, and mortality was modeled using Cox proportional hazards regression, adjusting for demographic and clinical covariates. Natural direct effects, natural indirect effects (NIE), and total effects were estimated using the mediation package in R (version 4.5.0), with 1000 nonparametric bootstrap replications to obtain 95% CIs. Effect estimates for the natural direct effects and NIE are reported on the log-hazard scale, consistent with the Cox proportional hazards model. The proportion mediated was calculated as the ratio of the NIE to the total effect (NIE/total effect). For each kidney function measure, the reported mediation proportion corresponds to the contrast between the highest category (e.g., ACR > 300 mg/g, CKD present) and the reference category. As sensitivity analyses, we repeated all Cox proportional hazards regression and mediation models with additional adjustment for body mass index (BMI) and smoking status (never, former, and current). In these analyses, BMI and smoking were treated as potential confounders rather than as mediators. This approach allows us to assess the robustness of the primary associations after accounting for these important lifestyle and metabolic factors. We did not treat them as mediators due to the cross-sectional nature of the exposure and mediator assessment, which precludes establishing temporal precedence. All analyses were performed using R software, version 4.2.1 (R Foundation for Statistical Computing). Statistical significance was set at a 2-sided *P* < .05.

## 3. Results

### 3.1. Demographic characteristics of the study population

Table 1 presents the descriptive characteristics of the study population stratified by frailty status. Among 9079 individuals, the prevalence of frailty in this cohort was 15.7%, which is in line with other studies using the same study population.^[24]^ Frail participants were more likely to be older, female, and have lower educational attainment compared to their non-frail counterparts (all *P* < .001). They also exhibited a higher prevalence of CKD, diabetes, and hypertension. Regarding kidney function measures, frail individuals had lower mean eGFR and higher mean ACR compared to non-frail participants.

**Table 1:** Descriptive characteristics of participants by frailty status.

	Overall (N = 9079)	Non-frail (N = 7658)	Frail (N = 1421)	*P* value
Age, N (%)				<.001
45–54	978 (10.8)	710 (9.3)	268 (18.9)	
55–64	2864 (31.5)	2393 (31.2)	471 (33.1)	
65–74	3011 (33.2)	2678 (35.0)	333 (23.4)	
75 and above	2226 (24.5)	1877 (24.5)	349 (24.6)	
Gender, N (%)				<.001
Male	4592 (50.6)	4021 (52.5)	571 (40.2)	
Female	4487 (49.4)	3637 (47.5)	850 (59.8)	
Race, N (%)				.008
Mexican American	1080 (11.9)	922 (12.0)	158 (11.1)	
Other Hispanic	966 (10.6)	807 (10.5)	159 (11.2)	
Non-Hispanic White	4406 (48.5)	3733 (48.7)	673 (47.4)	
Non-Hispanic Black	1885 (20.8)	1548 (20.2)	337 (23.7)	
Other race	742 (8.2)	648 (8.5)	94 (6.6)	
Education, N (%)				<.001
Less than high school education attainment	2600 (28.6)	2118 (27.7)	482 (33.9)	
High school graduate	2213 (24.4)	1836 (24.0)	377 (26.5)	
Has more than a high school education	4266 (47.0)	3704 (48.4)	562 (39.5)	
Marital status, N (%)				<.001
Married or partnered	5301 (58.4)	4645 (60.7)	656 (46.2)	
Not married or partnered	3778 (41.6)	3013 (39.3)	765 (53.8)	
eGFR, mean (SD), (mL/min/1.73 m^2^)	75.18 (19.62)	75.52 (18.97)	73.36 (22.70)	<.001
eGFR groups, N (%)				
<60	1965 (21.6)	1587 (20.7)	378 (26.6)	<.001
60–89	4812 (53.0)	4140 (54.1)	672 (47.3)	
>90	2302 (25.4)	1931 (25.2)	371 (26.1)	
ACR, mean (SD) (mg/g)	68.44 (411.62)	59.25 (376.99)	117.94 (560.28)	<.001
ACR groups, N (%)				<.001
<30	7334 (80.8)	6313 (82.4)	1021 (71.9)	
30–300	1413 (15.6)	1106 (14.4)	307 (21.6)	
>300	332 (3.7)	239 (3.1)	93 (6.5)	
CKD, N (%)				<.001
No	6026 (66.4)	5220 (68.2)	806 (56.7)	
Yes	3053 (33.6)	2438 (31.8)	615 (43.3)	
HDL, mean (SD) (mg/dL)	53.79 (16.62)	54.06 (16.44)	52.35 (17.52)	<.001
TC, mean (SD) (mg/dL)	194.28 (43.74)	194.69 (43.01)	192.04 (47.43)	.036
Diabetes, n (%)				<.001
No	6913 (76.1)	5957 (77.8)	956 (67.3)	
Yes	2166 (23.9)	1701 (22.2)	465 (32.7)	
Hypertension, n (%)				<.001
No	3689 (40.6)	3279 (42.8)	410 (28.9)	
Yes	5390 (59.4)	4379 (57.2)	1011 (71.1)	
Mortality, n (%)				<.001
Assumed alive	7370 (81.2)	6322 (82.6)	1048 (73.8)	
Assumed deceased	1709 (18.8)	1336 (17.4)	373 (26.2)	
Follow-up time, median (IQR), month	77.0 (35.0–117.0)	85.0 (37.0–119.0)	51.0 (30.0–106.0)	<.001

* Abbreviations: ACR = albumin-to-creatinine ratio, CKD = chronic kidney disease, eGFR = estimates of glomerular filtration rate, HDL = high-density lipoprotein, TC = total cholesterol.

### 3.2. Associations between kidney function and frailty

**Figure 2.**
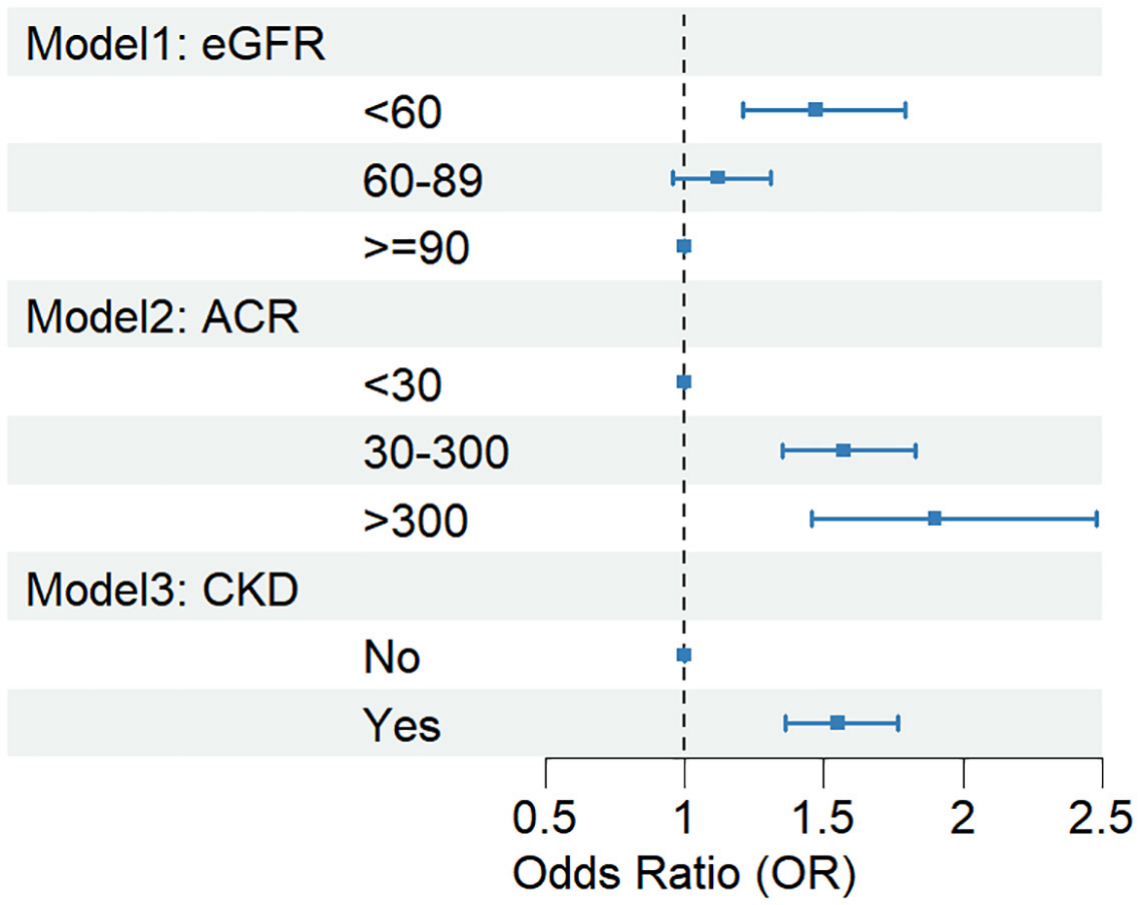
Relationships between kidney function measures and frailty. All models were adjusted for age, sex, educational level, race, marital status, HDL, TC, diabetes, and hypertension. Error bars represent 95% confidence intervals. Statistical significance was defined as *P* < .05 for all comparisons. ACR = albumin-to-creatinine ratio, CKD = chronic kidney disease, eGFR = estimated glomerular filtration rate, HDL = high-density lipoprotein cholesterol, TC = total cholesterol.

Figure 2 and Table S1, Supplemental Digital Content 1 illustrate the relationships between kidney function measures and frailty, adjusted for age, sex, educational level, race, marital status, HDL, TC, diabetes, and hypertension. Compared to participants with eGFR ≥ 90 mL/min/1.73 m^2^, those with lower eGFR demonstrated significantly higher odds of frailty. Conversely, elevated ACR was associated with increased odds of frailty. Relative to individuals with ACR < 30 mg/g, those with higher ACR levels exhibited progressively greater odds of frailty (30–300 mg/g: odds ratio [OR] = 1.57, 95% CI = 1.35–1.83, *P* < .001; > 300 mg/g: OR = 1.90, 95% CI = 1.46–2.48, *P* < .001). Furthermore, the presence of CKD was linked to a 55% higher likelihood of frailty compared to its absence (OR = 1.55, 95% CI = 1.36–1.77, *P* < .001).

Furthermore, we examined eGFR and ACR as continuous variables to explore their associations with frailty. A 10 mL/min/1.73 m^2^ decline in eGFR was associated with an 8% increase in the odds of frailty (OR = 1.08, 95% CI = 1.05–1.11, *P* < .001). Similarly, a unit increase in log-transformed ACR was associated with 25% higher odds of frailty (OR = 1.25, 95% CI = 1.18–1.32, *P* < .001).

### 3.3. Kidney function, frailty, and mortality

**Figure 3.**
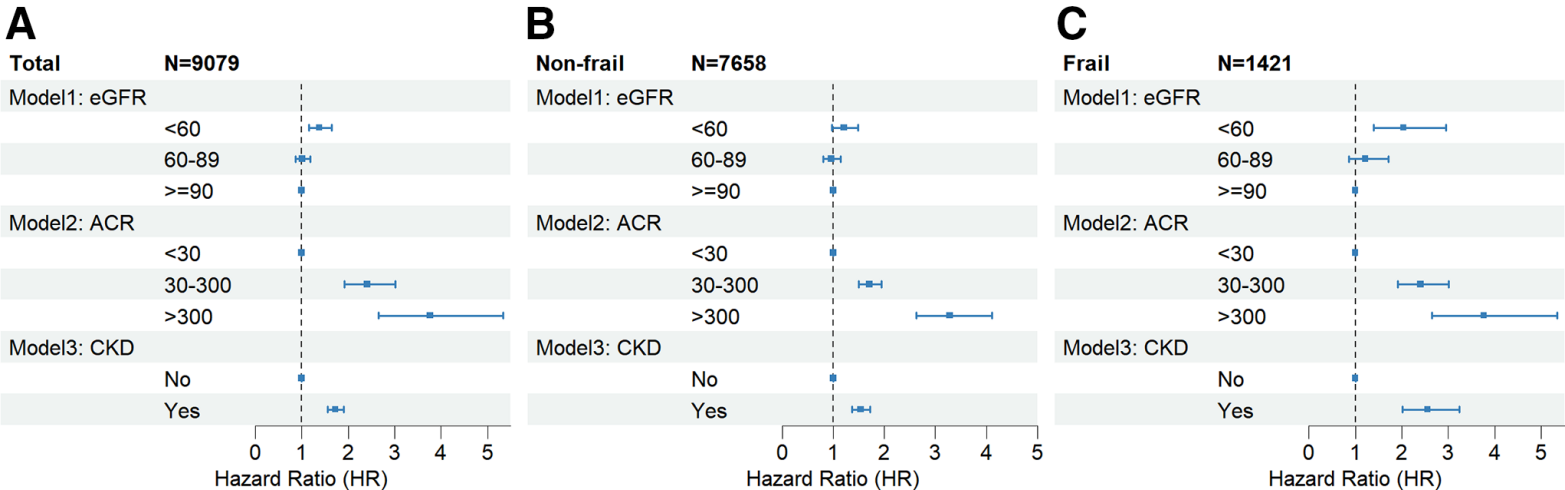
Relations between kidney function measures and mortality stratified by frailty. (A) Total sample; (B) Non-frail participants; (C) Frail participants. All models were adjusted for age, sex, educational level, race, marital status, HDL, TC, diabetes, and hypertension. In the total sample, the model was additionally adjusted for frailty. Stratified analyses are descriptive only, and no formal interaction term was tested. Error bars represent 95% confidence intervals. Statistical significance was defined as *P* < .05 for all comparisons. ACR = albumin-to-creatinine ratio, CKD = chronic kidney disease, eGFR = estimated glomerular filtration rate, HDL = high-density lipoprotein cholesterol, TC = total cholesterol.

The proportional hazards assumption was verified using Schoenfeld residual tests. The global test showed no significant violation of the assumption (χ^2^ = 14.32, *P* = .082). Individual tests for each covariate were also nonsignificant (all *P* > .05), supporting the validity of the Cox proportional hazards models. Over a median follow-up of 77 months (interquartile range: 35–117 months), 1709 deaths (18.8%) occurred. Frail individuals had a higher mortality rate compared to non-frail participants (*P* < .001). Figure 3A and Table S1, Supplemental Digital Content 1, depict the associations between kidney function measures and mortality in the total sample. In the fully adjusted model, impaired kidney function was associated with increased mortality risk. Compared to participants with eGFR ≥ 90 mL/min/1.73 m^2^, those with lower eGFR demonstrated significantly higher mortality risk (<60 mL/min/1.73 m^2^: HR = 1.47, 95% CI = 1.15–1.65, *P* < .001), indicating a 47% higher risk of death. Elevated ACR was linked to increased mortality risk (30–300 mg/g: HR = 1.87, 95% CI = 1.67–2.09, *P* < .001; > 300 mg/g: HR = 3.44, 95% CI = 2.85–4.15, *P* < .001). The presence of CKD was associated with a 72% higher mortality risk (HR = 1.72, 95% CI = 1.55–1.91, *P* < .001). In sensitivity analyses additionally adjusting for BMI and smoking status (Table S2, Supplemental Digital Content 2), most estimates remained stable in terms of direction, magnitude, and statistical significance. However, 1 notable change was observed: in the non-frail subgroup, the association between eGFR < 60 mL/min/1.73 m^2^ (vs ≥90) and mortality shifted from borderline nonsignificant in the primary analysis (HR = 1.301, 95% CI = 0.980–1.720, *P* = .071) to statistically significant after adjustment for BMI and smoking (HR = 1.280, 95% CI = 1.040–1.570, *P* = .020). This suggests that in non-frail individuals, BMI and smoking status may have acted as confounders in the association between reduced eGFR and mortality. Analyses stratified by frailty status (Figs. 3B and C) revealed that the magnitude of the associations between kidney function measures and mortality differed across groups. Among non-frail participants, elevated ACR was linked to increased mortality risk (30–300 mg/g: HR = 1.71, 95% CI = 1.50–1.95, *P* < .001; >300 mg/g: HR = 3.29, 95% CI = 2.63–4.11, *P* < .001). The presence of CKD was associated with higher mortality risk (HR = 1.54, 95% CI = 1.37–1.73, *P* < .001). No significant association between eGFR and mortality risk was found among non-frail individuals. Among frail participants, similar patterns were observed, with lower eGFR levels associated with a significantly higher mortality risk and elevated ACR being associated with increased mortality risk (30–300 mg/g: HR = 2.41, 95% CI = 1.92–3.02, *P* < .001; >300 mg/g: HR = 3.77, 95% CI = 2.66–5.35, *P* < .001). Similarly, the HR for CKD presence was higher in frail participants (HR = 2.56, 95% CI = 2.02–3.24, *P* < .001) compared with non-frail participants. However, these subgroup comparisons are descriptive only and, as noted in the Methods section, do not constitute a formal test of statistical interaction. In addition to the categorical analysis, eGFR and uACR were analyzed as continuous variables to provide further insights. The continuous analysis revealed consistent trends with the categorical results. A 10 mL/min/1.73 m^2^ decline in eGFR was associated with a 12% increase in mortality risk (HR = 1.12, 95% CI = 1.08–1.16, *P* < .001). Similarly, a unit increase in log-transformed uACR was associated with a 34% higher mortality risk (HR = 1.34, 95% CI = 1.25–1.43, *P* < .001). These findings support the robustness of the associations observed in the categorical analyses.

### 3.4. Mediation analysis

**Figure 4.**
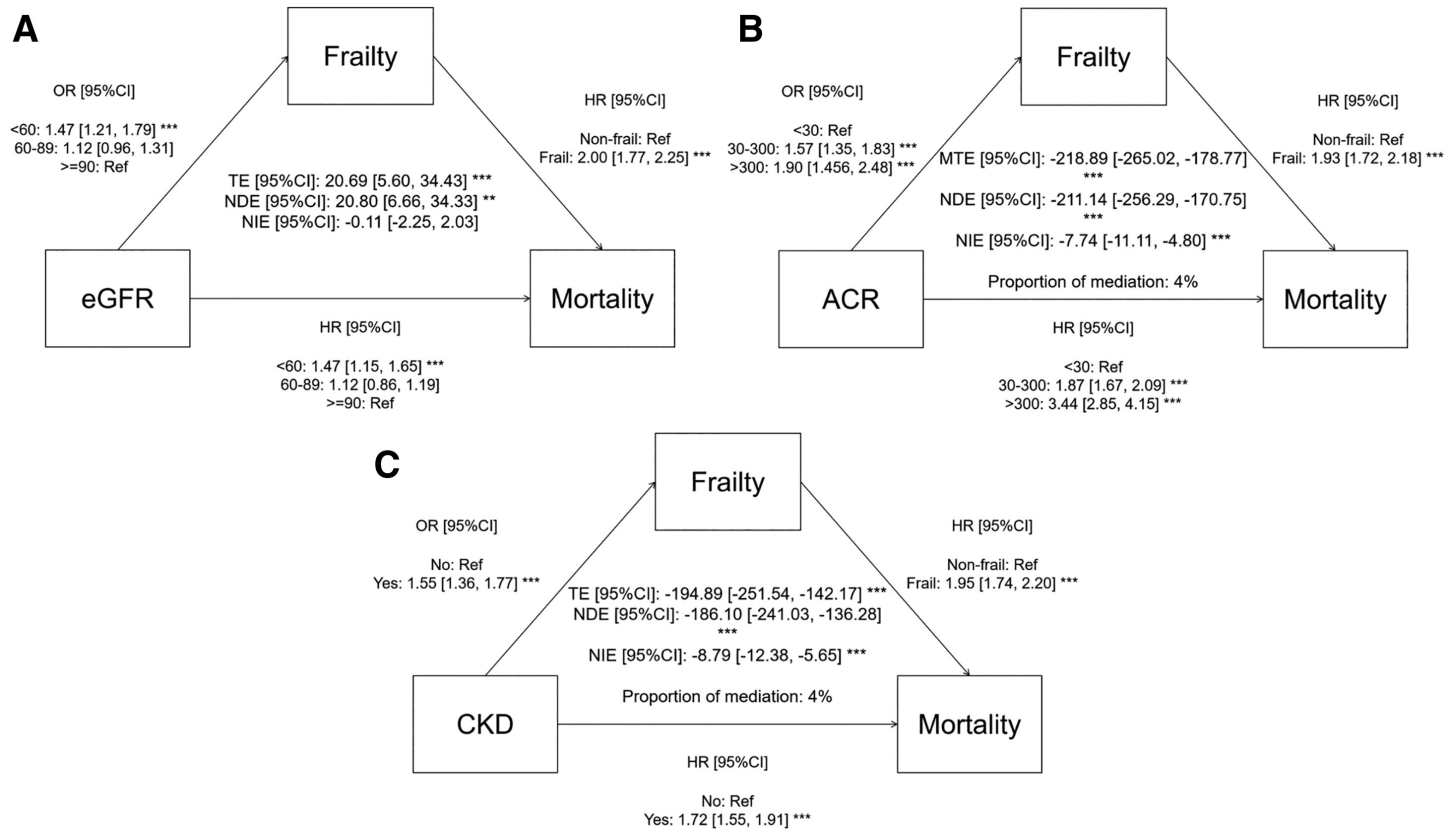
The mediating role of frailty in the relationship between kidney function measures and mortality. (A) eGFR; (B) ACR; (C) CKD status. Models adjusted for age, sex, educational level, race, marital status, HDL, TC, diabetes, and hypertension. The MTE, NDE, and NIE of kidney function measures on mortality were derived using the parameters obtained from multivariable logistic models and Cox models. In these models, frailty was regressed on kidney function measures. Additionally, parameters from Cox models, which factored in mortality, were regressed on kidney function measures and frailty. Within the counterfactual framework, the NIE is utilized to discern the indirect impact of kidney function measures on mortality through frailty. Mediation effect estimates are reported on the log-hazard scale, consistent with the Cox proportional hazards model. The proportion mediated was calculated as the natural indirect effect (NIE) divided by the total effect. For ACR and CKD, the reported estimates correspond to the contrast between the highest category (ACR > 300 mg/g; CKD present) and the reference category. For eGFR, the contrast is between <60 and ≥90 mL/min/1.73 m^2^. Error bars represent 95% confidence intervals obtained from 1000 bootstrap replications. * *P* < .05; ** *P* < .01; *** *P* < .001. ACR = albumin-to-creatinine ratio, CKD = chronic kidney disease, eGFR = estimated glomerular filtration rate, HDL = high-density lipoprotein cholesterol, HR = hazard ratio, MTE = mediation total effect, NDE = natural direct effect, NIE = natural indirect effect, OR = odds ratio, TC = total cholesterol.

Figure 4 and Table S3, Supplemental Digital Content 3 present the results of the mediation decomposition analysis examining whether baseline frailty statistically accounted for part of the association between kidney function measures and mortality. All mediation effect estimates are reported on the log-hazard scale, consistent with the Cox model. Frailty accounted for a modest but statistically significant proportion of the association between ACR and mortality. For the contrast between the highest ACR category (>300 mg/g) and the reference category (<30 mg/g), the NIE was − 7.74 (95% CI = −11.11 to −4.80, *P* < .001), and the proportion mediated was 4% (95% CI = 2%–5%). Similarly, for CKD presence versus absence, frailty mediated 4% of the total association (NIE = −8.79, 95% CI = −12.38 to −5.65, *P* < .001; proportion mediated = 4%, 95% CI = 3%–6%). In contrast, no significant mediation effect was observed for eGFR. For the contrast between eGFR < 60 mL/min/1.73 m^2^ and the reference category (≥90 mL/min/1.73 m^2^), the NIE was −0.11 (95% CI = −2.25 to 2.03, *P* = .868), with a proportion mediated of −1% (95% CI: −21% to 11%). These findings suggest that frailty statistically contributes to the association between impaired kidney function (as measured by ACR and CKD status) and mortality; however, the majority of the association operates through pathways independent of frailty. Sensitivity analyses additionally adjusting for BMI and smoking status yielded materially unchanged results (Table S4, Supplemental Digital Content 4).

## 4. Discussion

In this large cohort of middle-aged and older adults from NHANES 2007 to 2018, we found significant associations between measures of kidney function and all-cause mortality, with frailty playing a significant mediating role. Higher eGFR was associated with lower mortality risk, while elevated ACR and the presence of CKD were linked to increased mortality risk. Importantly, mortality associations with kidney function were descriptively stronger in frail participants. Mediation analyses further showed that frailty mediated a modest but statistically significant proportion (4%) of the association between ACR/CKD and mortality. These findings, reported on the log-hazard scale, suggest that frailty represents 1 potential pathway through which these markers of kidney damage may influence survival. However, the majority of the association (96%) operated through pathways independent of frailty, highlighting the multifactorial nature of the relationship between kidney dysfunction and mortality. No significant mediation was observed for eGFR, suggesting that the pathways linking reduced filtration rate to mortality may be less dependent on the frailty syndrome as defined in this study.

Our study revealed a significant association between impaired kidney function and increased frailty prevalence, consistent with emerging evidence in this field. Shi et al demonstrated a dose–response relationship between declining GFR and frailty likelihood in older adults, suggesting that even mild renal impairment may impact overall health.^[25]^ This relationship has been documented globally. In Japan, Inoue et al corroborated these findings, showing that individuals with CKD had a higher frailty prevalence compared to those without CKD, with even modest reductions in kidney function associated with elevated frailty risk.^[26]^ Similarly, an independent association between low-grade albuminuria and frailty was identified in community-dwelling middle-aged and older adults in Taiwan, suggesting albuminuria may serve as an early indicator of both kidney dysfunction and frailty risk.^[27]^ Furthermore, European longitudinal data from the Berlin Initiative Study showed that older adults with advanced CKD or significant albuminuria had double the risk of frailty progression.^[12]^ These findings underscore the importance of routine monitoring of kidney function parameters, including GFR and albuminuria, which may facilitate early identification of individuals at heightened risk for frailty.

Our findings suggest that kidney function and frailty are closely linked. The association between impaired kidney function and all-cause mortality was more pronounced among frail individuals, and frailty also mediated part of this relationship. Consistent with previous research, we observed that impaired kidney function was associated with increased mortality risk, with this association being particularly pronounced among frail participants. Frailty is characterized by diminished physiological reserves and increased vulnerability to stressors.^[7,28]^ Associations between impaired kidney function and mortality were stronger among frail individuals. Merchant and Vathsala reported that frail individuals with CKD exhibit a reduced capacity to compensate for the physiological challenges posed by declining renal function, resulting in elevated mortality risk.^[6]^ The impact of frailty on the relationship between kidney function and all-cause mortality is further complicated by its frequent coexistence with multiple chronic conditions. In frail individuals, the co-occurrence of CKD with other comorbidities, such as cardiovascular disease, diabetes, and osteoporosis, creates a complex clinical picture that significantly elevates mortality risk.^[29]^ This cumulative burden of multiple comorbidities in frail individuals suggests that even modest declines in kidney function may precipitate fatal outcomes, consistent with stronger mortality associations among frail participants.. The sensitivity analysis adjusting for BMI and smoking revealed that in non-frail individuals, the association between eGFR < 60 and mortality became statistically significant only after accounting for these factors. This has 2 clinical implications: First, the detrimental effect of reduced kidney function on survival may be confounded by BMI and smoking in non-frail populations. Second, assessing BMI and smoking status alongside eGFR in non-frail patients may improve risk identification, as the association between kidney dysfunction and mortality becomes clearer when these factors are considered. The stability of most other estimates after adjustment reinforces the robustness of the primary findings. The specificity of this change to the non-frail subgroup and to eGFR suggests that the confounding role of BMI and smoking may differ by frailty status and by the specific measure of kidney function.

Our study also reveals that frailty serves as a pathway through which impaired kidney function influences mortality risk. This association can be understood through multiple interconnected mechanisms, including inflammation, sarcopenia, and hormonal dysregulation. Impaired kidney function is associated with elevated levels of pro-inflammatory cytokines, including interleukin-6 and tumor necrosis factor-alpha, which not only contribute to the progression of kidney damage but also promote systemic inflammation.^[10,11]^ This inflammatory milieu is known to accelerate muscle wasting, cognitive decline, and overall physical deterioration, hallmarks of the frailty syndrome.^[30]^ Moreover, sarcopenia, the progressive loss of skeletal muscle mass and function, represents another critical mechanism linking CKD and frailty. CKD patients experience accelerated muscle protein breakdown through multiple pathways, including metabolic acidosis, insulin resistance, and reduced physical activity.^[31]^ Additionally, hormonal dysregulation plays a significant role, with CKD patients exhibiting decreased levels of anabolic hormones such as testosterone and insulin-like growth factor-1, further compromising muscle protein synthesis and contributing to frailty development. These mechanisms are associated with both worsening kidney function and progressive frailty, establishing a detrimental feedback loop where each condition exacerbates the other. This relationship significantly elevates mortality risk, as the combined physiological burden may overwhelm compensatory mechanisms when faced with additional stressors such as infections or cardiovascular events.^[7,32]^ Additionally, nutritional interventions and pharmacological approaches targeting inflammation show promise in mitigating the progression of both frailty and CKD. Future research should investigate the efficacy of multimodal interventions that simultaneously address inflammation, sarcopenia, and hormonal dysregulation in this vulnerable population. These findings underscore the importance of considering frailty status when assessing mortality risk in individuals with impaired kidney function. The mediating role of frailty, together with the observed heterogeneity across frailty strata, highlights the need for comprehensive assessment and management strategies that consider frailty status in this vulnerable population.The clinical implications of our findings are substantial for CKD management. Integration of frailty screening into routine nephrology care could enable early identification of high-risk individuals and guide personalized treatment strategies. Simple, validated tools such as the Clinical Frailty Scale or the FRAIL scale could be implemented in outpatient settings with minimal time and resource requirements.^[33]^ For identified frail CKD patients, comprehensive geriatric assessment could inform multidisciplinary care plans incorporating nutritional optimization, structured exercise programs, medication review, and advance care planning.^[33]^ Recent evidence from international studies suggests that targeted interventions can modify frailty trajectories in CKD patients. Exercise programs tailored to frail individuals have demonstrated improvements in muscle strength and reduction in inflammatory markers, potentially mitigating the progression of both frailty and CKD.^[34–36]^ These findings underscore the importance of considering frailty status when assessing mortality risk in individuals with impaired kidney function, highlighting the need for comprehensive assessment and management strategies in this vulnerable population.

Our study has several notable strengths. We utilized publicly available data from a large, population-based cohort, collected under well-controlled and rigorous protocols, which enhances the generalizability of our findings. The incorporation of multiple kidney function measures allowed us to establish robust relationships among kidney function, frailty, and all-cause mortality. Additionally, our investigation into the role of frailty in the association between kidney function and mortality provides novel insights into this complex interplay. Nevertheless, several limitations warrant consideration. First, there is potential for selection bias, as individuals excluded due to missing frailty assessments or covariates may differ systematically from those included in the analysis. This could overrepresent healthier individuals and limit generalizability. Additionally, the higher proportion of younger frail participants may reflect real-world phenomena or limitations in frailty definitions. Future studies should address these issues through sensitivity analyses and improved data handling. Also, although NHANES employs a complex multistage probability sampling design, we did not apply sampling weights, strata, or primary sampling units in our regression and mediation analyses. This approach was appropriate for examining internal associations within the analytic sample – the primary inferential target of this study. However, it limits the generalizability of our findings to the broader U.S. population. The observed associations may not hold in populations with different demographic or clinical characteristics. Future studies aiming to produce nationally representative estimates should incorporate survey weights and design elements in accordance with CDC/NHANES analytic guidance. Because we did not include a kidney function × frailty interaction term in a single model, we cannot formally conclude effect modification. Observed differences across strata should be interpreted as suggestive heterogeneity. Cross-sectional regression models were used to evaluate the baseline associations between kidney function and frailty. The cross-sectional nature of the frailty analysis precludes causal inferences, and the dynamic nature of frailty, which evolves over time, is not captured. This limitation underscores the need for longitudinal studies that can track changes in frailty status and health-related behaviors over time. Also, inflammation is critical in the development of kidney disease, frailty, and mortality. However, markers like C-reactive protein and interleukin-6 and health behaviors like smoking status were not consistently available or collected across all NHANES 2007 to 2018 cycles, preventing their inclusion in our models. BMI and smoking were treated as confounders to test robustness. However, due to cross-sectional assessment, they could be mediators. If so, adjustment would underestimate the total effect. Thus, findings reflect robustness testing, not causal estimation. Longitudinal studies are needed to clarify temporal relationships. These limitations underscore the need for longitudinal studies to elucidate the temporal dynamics among kidney function, frailty, and mortality. Future research should also employ strategies to minimize data incompleteness and mitigate selection bias, such as multiple imputation techniques or sensitivity analyses. Despite these constraints, our findings provide valuable insights into the complex relationships among kidney function, frailty, and mortality risk in older adults, laying the groundwork for future investigations and potential clinical applications.

In conclusion, our findings demonstrate significant associations between kidney function, frailty, and mortality risk in US middle-aged and older adults. Frailty mediated a modest proportion (approximately 4%) of the association between ACR/CKD and mortality. Associations between impaired kidney function and mortality were descriptively stronger among frail individuals. These results highlight the need to incorporate frailty assessment into risk evaluation and management for individuals with kidney disease. Future research should use longitudinal designs to clarify temporal relationships and explore mechanisms linking kidney dysfunction, inflammation, and frailty to inform targeted interventions.

## Acknowledgments

We acknowledge the members of the National Center for Health Statistics of the Centers for Disease Control and Prevention and all participants of the National Health and Nutrition Examination Survey.

## Author contributions

**Conceptualization:** Baosai Lu, Chenming Zhao, Wei Li.

**Data curation:** Bin Li.

**Formal analysis:** Bin Li, Yalin Niu, Chenming Zhao.

**Investigation:** Yalin Niu.

**Software:** Yalin Niu.

**Supervision:** Baosai Lu, Yuewei Yin, Wei Li.

**Validation:** Baosai Lu, Yuewei Yin.

**Writing – original draft:** Bin Li, Chenming Zhao.

**Writing – review & editing:** Baosai Lu, Yuewei Yin, Chenming Zhao, Wei Li.








