# Supplementary material for: Frailty mediates the relationship between kidney function measures and all-cause mortality among middle-aged and older adults: Findings from stratified analysis
Source: Medicine (Baltimore). 2026 Jun 19;105(25):e49214. doi: 10.1097/MD.0000000000049214 (PMC13286490; doi:10.1097/MD.0000000000049214)
Supplement: Supplementary file 1 [file medi-105-e49214-s001.docx]

**Table S1. The associations between kidney function, frailty, and mortality.**

| **Variables** | |  | **Estimates** | **95% CI** | **P-value** |
| --- | --- | --- | --- | --- | --- |
| **The associations between kidney function and frailty** | | | | | |
| Categorical eGFR (ref: ≥ 90) | < 60 | | 1.474 | (1.213, 1.791) | <.001 |
|  | 60-89 | | 1.122 | (0.96, 1.311) | 0.147 |
| Continuous eGFR |  | | 0.991 | (0.988, 0.995) | <.001 |
| Categorical ACR (ref: < 30) | Moderately 30-300 | | 1.572 | (1.351, 1.83) | <.001 |
|  | Severely >300 | | 1.901 | (1.457, 2.48) | <.001 |
| Continuous ACR |  | | 1.002 | (1.000, 1.003) | 0.014 |
| CKD (ref: No) | YES | | 1.551 | (1.361, 1.767) | <.001 |
|  |  | |  |  |  |
| **The associations between kidney function and mortality** | | | | | |
| **Total sample (N=9,079)** | | |  |  |  |
| Categorical eGFR (ref: ≥ 90) | | < 60 | 1.474 | (1.152, 1.653) | <.001 |
|  | | 60-89 | 1.122 | (0.861, 1.192) | 0.875 |
| Continuous eGFR | |  | 0.990 | (0.987, 0.992) | <.001 |
| Categorical ACR (ref: < 30) | | Moderately 30-300 | 1.867 | (1.669, 2.089) | <.001 |
|  | | Severely >300 | 3.442 | (2.854, 4.151) | <.001 |
| Continuous ACR | |  | 1.003 | (1.002, 1.003) | 0.014 |
| CKD (ref: No) | | YES | 1.722 | (1.553, 1.910) | <.001 |
| **Non-frail sample (N=7,658)** | |  |  |  |  |
| Categorical eGFR (ref: ≥ 90) | | < 60 | 1.301 | (0.980, 1.720) | 0.071 |
|  | | 60-89 | 1.052 | (0.853, 1.290) | 0.660 |
| Continuous eGFR | |  | 0.992 | (0.989, 0.996) | <.001 |
| Categorical ACR (ref: < 30) | | Moderately 30-300 | 1.709 | (1.499, 1.949) | <.001 |
|  | | Severely >300 | 3.287 | (2.627, 4.113) | <.001 |
| Continuous ACR | |  | 1.003 | (1.002, 1.003) | <.001 |
| CKD (ref: No) | | YES | 1.537 | (1.367, 1.727) | <.001 |
| **Frail sample (N=1,421)** | |  |  |  |  |
| Categorical eGFR (ref: ≥ 90) | | < 60 | 2.035 | (1.403, 2.952) | <.001 |
|  | | 60-89 | 1.215 | (0.863, 1.711) | 0.265 |
| Continuous eGFR | |  | 0.983 | (0.978, 0.989) | <.001 |
| Categorical ACR (ref: < 30) | | Moderately 30-300 | 2.407 | (1.919, 3.018) | <.001 |
|  | | Severely >300 | 3.768 | (2.656, 5.346) | <.001 |
| Continuous ACR | |  | 1.003 | (1.002, 1.004) | <.001 |
| CKD (ref: No) | | YES | 2.557 | (2.020, 3.235) | <.001 |

*Abbreviations:* eGFR, estimated glomerular filtration rate (mL/min/1.73 m²); ACR, albumin-to-creatinine ratio (mg/g); CKD, chronic kidney disease; HR, hazard ratio; CI, confidence interval; BMI, body mass index.

*Note:* All models were adjusted for age, sex, race/ethnicity, educational attainment, marital status, HDL cholesterol, total cholesterol, diabetes, and hypertension. In the total sample, models were additionally adjusted for frailty status.

Reference categories: eGFR ≥ 90 mL/min/1.73 m²; ACR < 30 mg/g; CKD = No.

p-values: All reported p-values are category-specific Wald test p-values from the fully adjusted Cox proportional hazards models. For categorical variables, the p-value tests the null hypothesis that the hazard ratio for that specific category (compared to the reference) equals 1.0.

Continuous variables: For continuous eGFR and ACR, the HR represents the change in mortality risk per 1-unit increase in the variable. ACR was log-transformed in regression models to satisfy normality assumptions; the HR shown corresponds to the original scale for interpretability.

Verification: All HRs, 95% CIs, and p-values in each row have been verified against the original R model output files to ensure consistency.
