# Supplementary material for: Frailty mediates the relationship between kidney function measures and all-cause mortality among middle-aged and older adults: Findings from stratified analysis
Source: Medicine (Baltimore). 2026 Jun 19;105(25):e49214. doi: 10.1097/MD.0000000000049214 (PMC13286490; doi:10.1097/MD.0000000000049214)
Supplement: Supplementary file 2 [file medi-105-e49214-s002.docx]

**Table S2. Sensitivity analysis of the associations between kidney function measures and all-cause mortality, additionally adjusted for BMI and smoking status**

| **Kidney function measure** | **Category** | **Estimates** | **95% CI** | **P-value** |
| --- | --- | --- | --- | --- |
| ***Total sample*** |  |  |  |  |
| Categorical eGFR  (ref: ≥ 90) | < 60 | 1.47 | (1.23, 1.76) | <0.001 |
|  | 60-89 | 1.06 | (0.90, 1.24) | 0.500 |
| Categorical ACR  (ref: < 30) | Moderately 30-300 | 1.81 | (1.62, 2.03) | <0.001 |
|  | Severely >300 | 3.32 | (2.75, 4.01) | <0.001 |
| CKD (ref: No) | Yes | 1.72 | (1.55, 1.91) | <0.001 |
| ***Non-frail*** |  |  |  |  |
| Categorical eGFR  (ref: ≥ 90) | < 60 | 1.28 | (1.04, 1.57) | 0.020 |
|  | 60-89 | 1.00 | (0.84, 1.21) | 0.958 |
| Categorical ACR  (ref: < 30) | Moderately 30-300 | 1.66 | (1.46, 1.90) | <0.001 |
|  | Severely >300 | 3.19 | (2.55, 3.99) | <0.001 |
| CKD (ref: No) | Yes | 1.54 | (1.37, 1.73) | <0.001 |
| ***Frail*** |  |  |  |  |
| Categorical eGFR  (ref: ≥ 90) | < 60 | 2.23 | (1.53, 3.23) | <0.001 |
|  | 60-89 | 1.23 | (0.88, 1.74) | 0.229 |
| Categorical ACR  (ref: < 30) | Moderately 30-300 | 2.32 | (1.85, 2.91) | <0.001 |
|  | Severely >300 | 3.65 | (2.57, 5.18) | <0.001 |
| CKD (ref: No) | Yes | 2.58 | (2.04, 3.26) | <0.001 |
|  |  |  |  |  |

*Abbreviations:* eGFR, estimated glomerular filtration rate (mL/min/1.73 m²); ACR, albumin-to-creatinine ratio (mg/g); CKD, chronic kidney disease; HR, hazard ratio; CI, confidence interval; BMI, body mass index.

*Note:* All models were adjusted for age, sex, race/ethnicity, educational attainment, marital status, HDL cholesterol, total cholesterol, diabetes, hypertension, BMI, and smoking status. In the total sample, models were additionally adjusted for frailty status. In these sensitivity analyses, BMI and smoking were treated as potential confounders rather than as mediators. This approach tests the robustness of primary associations after accounting for these lifestyle and metabolic factors.

Reference categories: eGFR ≥ 90 mL/min/1.73 m²; ACR < 30 mg/g; CKD = No.

p-values: All reported p-values are category-specific Wald test p-values from the fully adjusted Cox proportional hazards models.

Verification: All estimates have been verified against the original R model output files.
