# Supplementary material for: Frailty mediates the relationship between kidney function measures and all-cause mortality among middle-aged and older adults: Findings from stratified analysis
Source: Medicine (Baltimore). 2026 Jun 19;105(25):e49214. doi: 10.1097/MD.0000000000049214 (PMC13286490; doi:10.1097/MD.0000000000049214)
Supplement: Supplementary file 3 [file medi-105-e49214-s003.docx]

**Table S3. The association between categorical eGFR/ACR/CKD and frailty.**

|  | **Estimate** | **95% CI** | ***P*-value** |
| --- | --- | --- | --- |
| **Predictor: eGFR** |  |  |  |
| ACME (control) | -0.11 | (-2.20, 1.96) | 0.868 |
| ACME (treated) | -0.11 | (-2.30, 2.09) | 0.868 |
| ADE (control) | 20.80 | (6.68. 34.32) | 0.004 |
| ADE (treated) | 20.79 | (6.64, 34.35) | 0.004 |
| Total Effect | 20.69 | (5.60.34.43) | <.001 |
| Prop. Mediated (control) | -0.01 | (-0.20, 0.11) | 0.868 |
| Prop. Mediated (treated) | -0.01 | (-0.21, 0.12) | 0.868 |
| ACME (average) | -0.11 | (-2.25, 2.03) | 0.868 |
| ADE (average) | 20.80 | (6.66, 34.33) | 0.004 |
| Prop. Mediated (average) | -0.01 | (-0.21, 0.11) | 0.868 |
| **Predictor: ACR** |  |  |  |
| ACME (control) | -9.32 | (-13.45, -5.79) | <.001 |
| ACME (treated) | -6.16 | (-8.71, -3.82) | <.001 |
| ADE (control) | -212.73 | (-258.34, -172.10) | <.001 |
| ADE (treated) | -209.56 | (-254.27, -169.69) | <.001 |
| Total Effect | -218.89 | (-265.02, -178.77) | <.001 |
| Prop. Mediated (control) | 0.04 | (0.03, 0.06) | <.001 |
| Prop. Mediated (treated) | 0.03 | (0.02, 0.04) | <.001 |
| ACME (average) | -7.74 | (-11.11, -4.80) | <.001 |
| ADE (average) | -211.14 | (-256.29, -170.75) | <.001 |
| Prop. Mediated (average) | 0.04 | (0.02, 0.05) | <.001 |
| **Predictor: CKD** |  |  |  |
| ACME (control) | -10.37 | (-14.72, -6.60) | <.001 |
| ACME (treated) | -7.20 | (-10.15, -4.64) | <.001 |
| ADE (control) | -187.69 | (-243.39, -137.05) | <.001 |
| ADE (treated) | -184.52 | (-238.78, -135.38) | <.001 |
| Total Effect | -194.89 | (-251.54, -142.17) | <.001 |
| Prop. Mediated (control) | 0.05 | (0.04, 0.07) | <.001 |
| Prop. Mediated (treated) | 0.04 | (0.02, 0.05) | <.001 |
| ACME (average) | -8.79 | (-12.38, -5.65) | <.001 |
| ADE (average) | -186.10 | (-241.03, -136.28) | <.001 |
| Prop. Mediated (average) | 0.04 | (0.03, 0.06) | <.001 |

*Abbreviations:* ACME, average causal mediation effect; ADE, average direct effect; eGFR, estimated glomerular filtration rate; ACR, albumin-to-creatinine ratio; CKD, chronic kidney disease; CI, confidence interval; BMI, body mass index.

*Note:* All models were adjusted for age, sex, race/ethnicity, educational attainment, marital status, HDL cholesterol, total cholesterol, diabetes, hypertension. All effect estimates (ACME, ADE, Total Effect) are reported on the log hazard scale, consistent with the Cox proportional hazards model used for the outcome. The proportion mediated is calculated as ACME (average) / Total Effect. For ACR, the estimates correspond to the contrast between ACR >300 mg/g and the reference category (<30 mg/g). For CKD, the estimates correspond to the contrast between CKD present and CKD absent. For eGFR, the estimates correspond to the contrast between eGFR <60 mL/min/1.73 m² and the reference category (≥90 mL/min/1.73 m²). Confidence intervals were obtained from 1,000 non-parametric bootstrap replications.
