# Supplementary material for: Frailty mediates the relationship between kidney function measures and all-cause mortality among middle-aged and older adults: Findings from stratified analysis
Source: Medicine (Baltimore). 2026 Jun 19;105(25):e49214. doi: 10.1097/MD.0000000000049214 (PMC13286490; doi:10.1097/MD.0000000000049214)
Supplement: Supplementary file 4 [file medi-105-e49214-s004.docx]

**Table S4. Sensitivity analysis of the mediating role of frailty in the relationship between kidney function measures and all-cause mortality, additionally adjusted for BMI and smoking status.**

| **Predictor** | **Component** | **Estimate** | **95% CI** | ***P*-value** |
| --- | --- | --- | --- | --- |
| **eGFR (categorical)** | ACME (average) | -0.10 | (-2.39, 2.19) | 0.940 |
|  | ADE (average) | 19.34 | (3.61, 33.60) | 0.016 |
|  | Total effect | 19.24 | (3.11, 33.50) | 0.012 |
|  | Proportion mediated | -0.004 | (-0.22, 0.20) | 0.944 |
| **ACR (categorical)** | ACME (average) | -7.79 | (-11.40, -4.52) | <0.001 |
|  | ADE (average) | -206.77 | (-252.20, -162.93) | <0.001 |
|  | Total effect | -214.55 | (-261.56, -169.58) | <0.001 |
|  | Proportion mediated | 0.036 | (0.02, 0.05) | <0.001 |
| **CKD (binary)** | ACME (average) | -8.85 | (-12.87, -5.54) | <0.001 |
|  | ADE (average) | -194.90 | (-252.75, -148.55) | <0.001 |
|  | Total effect | -203.75 | (-263.32, -156.52) | <0.001 |
|  | Proportion mediated | 0.043 | (0.03, 0.07) | <0.001 |
|  |  |  |  |  |

*Abbreviations:* ACME, average causal mediation effect; ADE, average direct effect; eGFR, estimated glomerular filtration rate; ACR, albumin-to-creatinine ratio; CKD, chronic kidney disease; CI, confidence interval; BMI, body mass index.

*Note:* All models were adjusted for age, sex, race/ethnicity, educational attainment, marital status, HDL cholesterol, total cholesterol, diabetes, hypertension, BMI, and smoking status.
